# Data clustering methods for the determination of cerebral autoregulation functionality

**DOI:** 10.1007/s10877-015-9774-8

**Published:** 2015-09-16

**Authors:** Dean Montgomery, Paul S. Addison, Ulf Borg

**Affiliations:** 1 Respiratory and Monitoring Solutions, Medtronic, Technopole Centre, Edinburgh, EH26 0PJ UK; 2 Respiratory and Monitoring Solutions, Medtronic, 6135 Gunbarrel Avenue, Boulder, CO 80301 USA

**Keywords:** Cerebral autoregulation, NIRS, Clustering, k-means, Gaussian mixture models, COx

## Abstract

Cerebral blood flow is regulated over a range of systemic blood pressures through the cerebral autoregulation (CA) control mechanism. The COx measure based on near infrared spectroscopy (NIRS) has been proposed as a suitable technique for the analysis of CA as it is non-invasive and provides a simpler acquisition methodology than other methods. The COx method relies on data binning and thresholding to determine the change between intact and impaired autoregulation zones. In the work reported here we have developed a novel method of differentiating the intact and impaired CA blood pressure regimes using clustering methods on unbinned data. K-means and Gaussian mixture model algorithms were used to analyse a porcine data set. The determination of the lower limit of autoregulation (LLA) was compared to a traditional binned data approach. Good agreement was found between the methods. The work highlights the potential application of using data clustering tools in the monitoring of CA function.

## Introduction

### Cerebral autoregulation

Cerebral blood flow (CBF) is regulated over a range of systemic blood pressures by the cerebral autoregulation (CA) control mechanism which acts through complex myogenic, neurogenic, and metabolic mechanisms [1]. This range spans a zone of ‘intact’ autoregulation from the lower limit of autoregulation (LLA) to the upper limit of autoregulation (ULA). Unregulated flow, and therefore ‘impaired’ CA, exists at the extremes of blood pressure (i.e. below the LLA and above the ULA) where cerebral vasocontrol is no longer able to adequately change vascular resistance in response to further blood pressure changes.

A number of measures have been developed to determine the patient’s autoregulation status as it is important for the clinician to know whether the patient is operating in an intact or impaired zone and, if the latter, to take appropriate action as required. The correlation between cerebral perfusion pressure (CPP) and transcranial Doppler (TCD)-measured cerebral blood flow (CBF) may be quantified using linear regression to assess autoregulation in patients with neurological conditions. The mean velocity index, Mx, quantifies the degree of correlation between blood pressure (BP) [usually taken as mean arterial pressure (MAP)] and cerebral flow, with positive values associated with an uncontrolled pressure-driven flow regime and hence compromised cerebral haemodynamic function [2]. However, although non-invasive, limitations of TCD include the need for frequency transponder repositioning and the inability to obtain a transcranial window in some patients [3].

### The COx measure

**Fig. 1:**
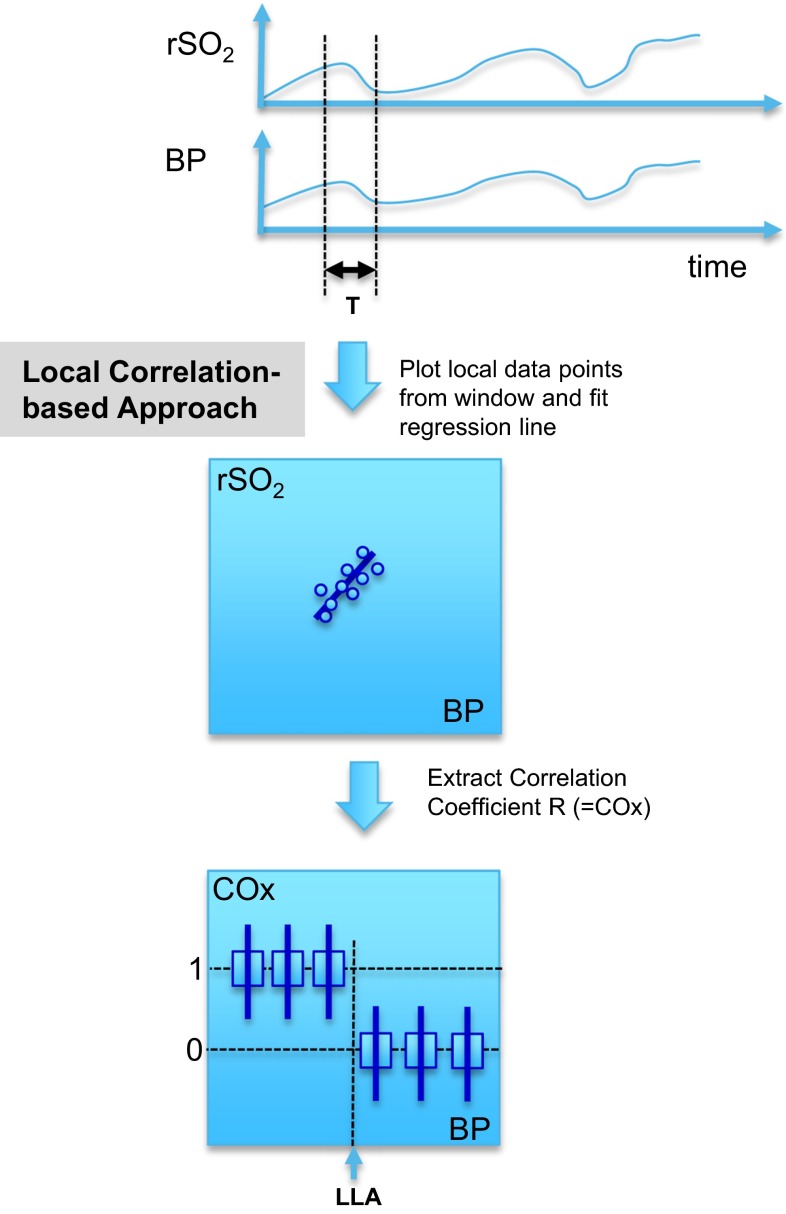
Schematic of the different methods for determining the LLA

The COx index has been developed over recent years in an attempt to derive a non-invasive, near infrared spectroscopy (NIRS)-based parameter for autoregulation [4, 5] that is a proxy for Mx. In addition to being non-invasive, NIRS is continuous, does not require the same level of caregiver manipulation associated with cerebral blood flow (CBF) measurement and the signal is more artefact-free than trans-cranial Doppler (TCD) waveforms [6]. Figure 1 illustrates the method for computing COx. The rSO_2_ signal and a blood pressure signal (mean arterial pressure) are acquired over time (top plot). An analysis window of period, T, runs across the signals and the rSO_2_ and BP data within the window are plotted against each other (middle left plot). The window must be of sufficient length to capture the characteristic periodicity of the physiological slow waves present in the blood pressure signal [7]. The linear regression line for this data is computed and the Pearson correlation coefficient obtained: this is the COx measure. The process repeats as the analysis window scans over the signals in incremental steps, resulting in a continuous COx measurement. The expectation is that a strong correlation exists between the two signals for regions of autoregulatory impairment, hence the COx values will be around unity. Regions of intact autoregulation, however, should produce no correlation between rSO_2_ and changes in blood pressure and hence we expect the mean correlation coefficient (COx) to be near zero. The COx signal is then binned in 5 mmHg increments according to the BP at the same time point. This is shown schematically in the lowest plot in the figure where the step change in the binned COx values indicates the LLA point. Note that there is an associated step up at the ULA at higher BPs (not shown in the figure for reasons of clarity). In practice, the binned data is generally noisy and a COx threshold between 0 and 1 is used to differentiate the correlating and non-correlating portions of the plot.

**Fig. 2:**
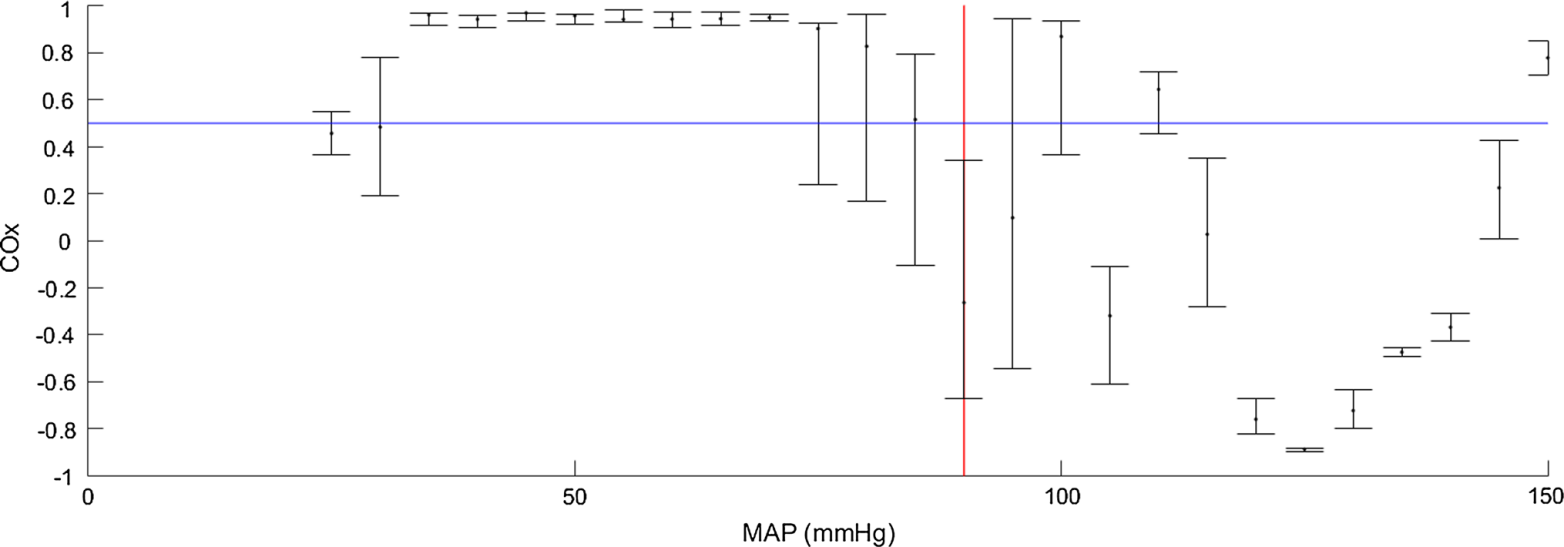
Binned COx Data against MAP. Binned COx data with a LLA (*red line*) determined by the crossing of a 0.5 threshold (*blue line*)

An example COx plot from an animal study is shown in Fig. 2. The data is shown in its traditional binned format where the COx values are collected in 5 mmHg bins showing the median value and interquartile ranges. In this example the data only spans the LLA. The impaired and intact regions may be clearly identified by eye. In practice the LLA point is identified through an algorithm which contains specific rules. For example, an LLA may be defined as the first median point below the threshold from left to right, where at least the previous two binned median values are above the threshold. More sophisticated criteria may also be used, for example a requirement that the determined LLA is a point belonging to a group of at least three consecutive values all below the threshold.

**Fig. 3:**
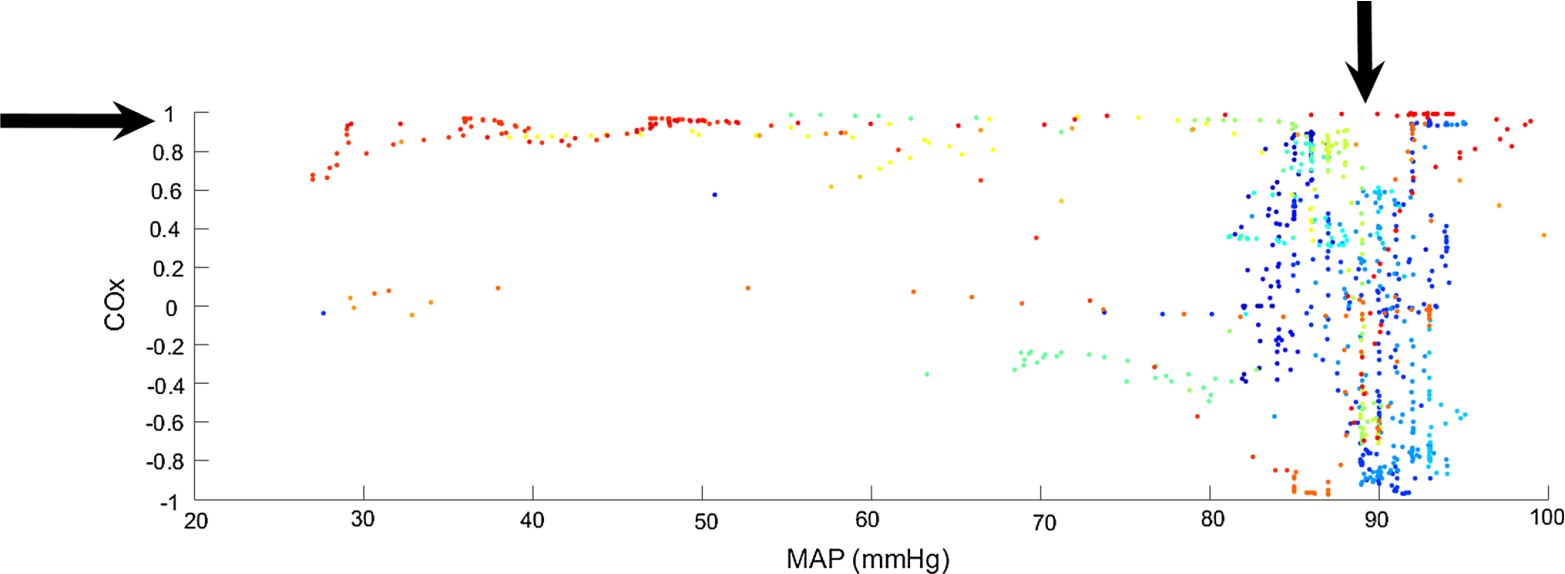
Unbinned COx Data. The *colours* of the points correspond to the different sections of the experimental protocol. See Fig. 4

The binned format, is traditional for viewing COx data [4, 6, 8, 9]. We have found that binning makes it difficult to produce a robust automated algorithm to determine LLAs due to the granular nature imposed on the bin-aggregated data. This issue is exacerbated early in a procedure when the complete picture of the COx plot has not yet built up or when blood pressure is relatively stable, and only a very few bins contain data points. However, we have noticed that by viewing the data in its raw format (i.e. unbinned), clear zones may be observed that correspond to the intact and impaired regions. This is shown in Fig. 3, where the individual data points of Fig. 2 are shown. Here we can clearly see a structure corresponding to the impaired and intact regions: the data in the impaired region is tightly clustered around a value of unity (horizontal arrow in the plot), whereas the intact region is distinctly spread out across a range from −1 to 1 (vertical arrow in the plot). This observation led us to study the potential of automated data clustering techniques for the determination of these regions and the boundary between them.

In the study reported here we sought to investigate the robustness of data clustering methods for the determination of the LLA using a historical data set from a porcine model which contained a number of interventions: hyper and hypo-ventilation, lung recruitment manoeuvres, acute hypoxia and haemorrhagic shock. The original purpose of this study was to determine NIRS-based saturation measurement performance over a series of different clinical scenarios. (Note that we do not determine the ULA in the work described here as the data set contained very few points at the higher values of BP required for such analysis.) In the work described herein, data clustering was performed using both the k-means and a Gaussian mixture model approaches [10].

## Methods

### Clinical study

A healthy porcine animal model was used of both sexes (four female/three male) with a mean weight of 16.4 kg (max/min = 22.2/13.4) and all approximately 12 weeks of age. The protocol was reviewed and approved by the PCRS Animal Care and Use Committee. The study was conducted in GLP like fashion in accordance with 21 CFR Part 58 at an Association for Assessment and Accreditation of Laboratory Animal Care (AAALAC) accredited site. The following standards in terms of appropriate use of animals for biomedical research and/or training were adhered to: The U.S. Animal Welfare Act amendment of 1976 (Title 9, Code of Federal Regulations, Chapter 1, Sub-chapter A, parts 1, 2 and 3) and the current U.S. National Institute of Health’s Guide for the Care and Use of Laboratory Animals published by the National Research Council. Isoflurane was used as the volatile anesthetic agent. Seven animals (N = 7) were studied. The porcine model is preferred since the thickness of the skull is similar to that of an adult human forehead. Additionally, the skin tone is similar to human skin and has similar light dispersion characteristics. NIRS sensors (INVOS SAFB-SM) were placed on the animals head between the ears. These were attached to the monitor [INVOS 5100C oximeter, 5100C-PA preamp unit. (INVOS, Boulder, CO)]. NIRS cerebral signals (both raw signals and the output rSO_2_ signal) and a blood pressure signal were collected. The animal was ventilated with a tidal volume of 6–8 ml/kg, FiO_2_ was adjusted to maintain 95 % arterial saturation and PEEP was 5 cm H_2_O. Respiratory rate was adjusted to maintain end-tidal CO_2_ between 38 and 45 mmHg. The study comprised a series of baseline periods followed by four discrete interventions as described below.

#### Baseline data

This was collected after all catheters were placed and the animal was in a steady state with stable haemodynamics (approximately 15 min post catheterisation) and between interventions.

#### Intervention 1: hyper and hypo-ventilation

- The animal was hyperventilated to an end-tidal CO_2_ level of approximately 30 mmHg by increasing the minute volume. Hyperventilation was confirmed from the arterial blood gas measurement. After 5 min of stable hyperventilation, arterial and venous blood gases were measured. Minute ventilation was then decreased back to pre-hyperventilation level and end-tidal CO_2_ was monitored to assess when the animal returned to baseline.
- The animal was then hypoventilated to an end-tidal CO_2_ level of approximately 55 mmHg by decreasing the minute ventilation. Hypoventilation was confirmed from arterial blood gas measurement. After 5 min of stable hypoventilation, arterial and venous blood gases were measured. Minute ventilation was then increased back to pre-hypoventilation level and end-tidal CO_2_ monitored to assess when the animal returned to baseline.

#### Intervention 2: lung recruitment maneuver

The animal was ventilated with baseline ventilator settings, and FiO_2_ was increased to 1.0. The ventilator was then changed to CPAP mode (or inspiratory hold) and the airway pressure was increased to 25 cm H_2_O for 40 s. Ventilation was again started at baseline settings for 5 min. After this the ventilator was changed to CPAP (or inspiratory hold) and the airway pressure increased to 35 cm H_2_O for 40 s. Ventilation was then started and the animal allowed to recover to baseline physiological values (cardiac output, SpO_2_ and end-tidal CO_2_).

#### Intervention 3: acute hypoxia

The animal was ventilated with baseline settings, and FiO_2_ was increased to 1.0. The FiO_2_ was changed rapidly to 0.08 to create acute hypoxia. When the animal reached the lowest level of SpO_2_, maintained for 2 min, blood samples were obtained from arterial and venous catheters, the FiO_2_ was rapidly increased to 1.0. When SpO_2_ returned to a 100 % plateau for 2 min blood samples were collected from arterial and venous catheters. This hypoxia procedure was then repeated before all settings were returned to baseline and the animal allowed to recover for 10 min.

#### Intervention 4: haemorrhagic shock

- The animal was ventilated with baseline settings and FiO_2_ of 1.0. Blood was removed through the central venous catheter until 60 % of the animal’s blood volume (blood volume in ml = 65*weight in kg) was removed in steps of 20 %.
- The animal was resuscitated with a combination of half of the shed blood and a balanced electrolyte solution in the same stepwise fashion until the mean arterial blood pressure returned to pre-haemorrhage levels.

**Fig. 4:**
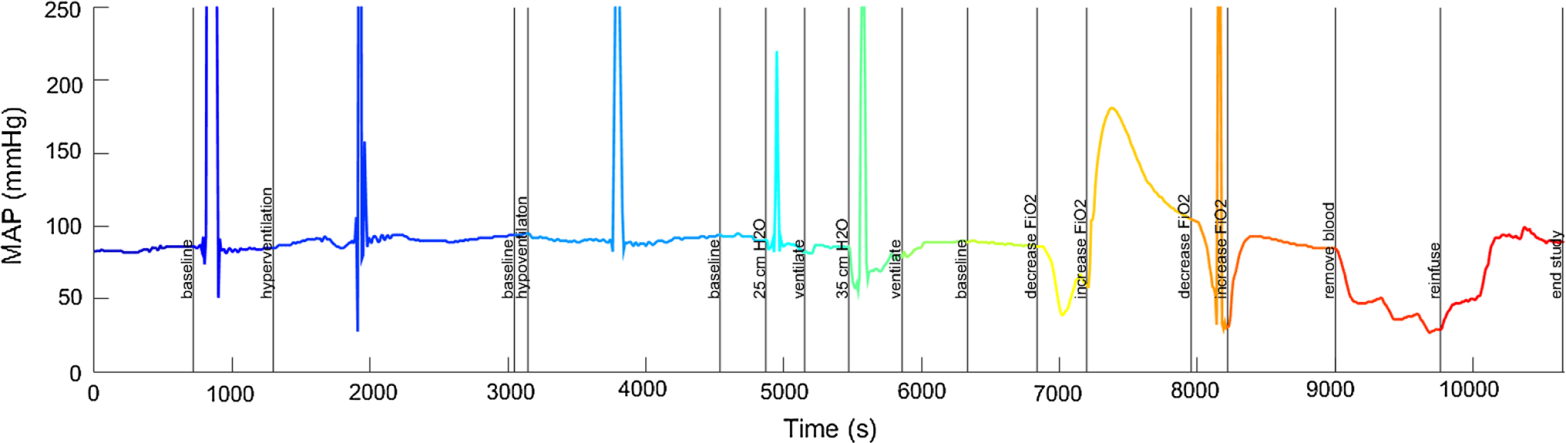
The blood pressure trace over the whole study for pig p0001. The *colour* of the *line* changes with each manoeuvre carried out during the experimental protocol. These *colours* also correspond to the *colours* of the points in Fig. 3

The behaviour of mean arterial pressure (MAP) during the study for one of the pigs is shown in Fig. 4, where the rapidly varying nature of the signal can be seen—note that the large spikes were due to blood draws and were ignored in the analysis. Although the pig blood pressures did reach substantially high values during the study procedure there was not enough time spent at the higher blood pressures to collect sufficient data for ULA determination. Hence this study focuses only on the LLA for each animal.

### Analysis

The COx autoregulation correlation metric was computed as described in Sect. 1 using a 300 s moving analysis window [11–13]. The window was run along the signals (MAP and rSO_2_) in a series of 10 s steps. The calculated metric values were binned in 5 mmHg blood pressure increments and the LLA point detected as the first point below a threshold of 0.5 using an automated algorithm which scans from left to right and setting a flag when two valid median binned points have been seen above the threshold. The next bin below the threshold is then marked as the LLA. Bins with fewer than five data points are marked as invalid. (Requiring at least two bins above the LLA prevents false positive LLA detection due to binned data outliers.) Note that in practice the threshold should be set somewhere between 0 and 1. This is because the expectation is for a value of unity for correlating regions (impaired CA) and a randomly spread out values of COx, with a mean of zero, for non-correlating regions (intact CA). In this work we use a threshold of 0.5. This is at the higher end of the values generally found in the literature which are 0.3 [14, 15], 0.4 [3, 16] and 0.5 [9, 17]. We argue that the value should be set high so as to distance the threshold from the noisy values tending to zero mean in the intact region and closer to the relatively tightly spread values at unity for the impaired region, hence we choose 0.5 for this work. The optimal value is a matter of debate and in practice would require a detailed parametric study employing a large data set to determine accurately. It is expected that different threshold values may be optimal for different demographic groups or for different areas of care.

The k-means and Gaussian mixture model methods were applied to the unbinned COx data for each animal. The algorithms used in the work here are described as follows.

#### The k-means algorithm

1. Choose k initial points as centroids (for example, here we used two random observations).
2. Calculate the distance from each point to each cluster centroid.
3. Individually assign each point to the centroid that decreases the sum of the within-cluster, sum-of-squares point-to-cluster-centroid distances.
4. Calculate the new centroids from the updated clusters.
5. Repeat 3–4 until convergence or max number of iterations reached.

The algorithm was run ten times to reduce the risk of being caught in local minima. The square of the Euclidean distance (for computational reasons the square root is not used) is employed as the distance metric and the COx and MAP were not rescaled prior to clustering.

#### The Gaussian mixture model algorithm

A number (N) of Gaussian distributions are fit to the data using an expectation maximization algorithm. This results in N Gaussian distributions for which the posterior probability of membership for each point can be calculated. A point is then said to be a member of the cluster for which it has the highest posterior probability. The whole algorithm is repeated in a similar manner to the k-means method in order to mitigate the effect of falling into local minima during the optimisation process.

Note that once the two clusters are found, the LLA is initially determined to be the minimum MAP value in the right hand cluster (the right hand cluster usually representing the region with a large spread in COx over a relatively narrow BP). An additional step to remove outliers from clusters was also applied before the final LLA determination. This required that all points in the right hand cluster that are further than two standard deviations away from the mean MAP for that cluster are discarded. This limits the LLA to the mean MAP of the cluster minus two standard deviations which we found has the effect of mitigating against obvious noise on the cluster plots.

## Results

**Fig. 5:**
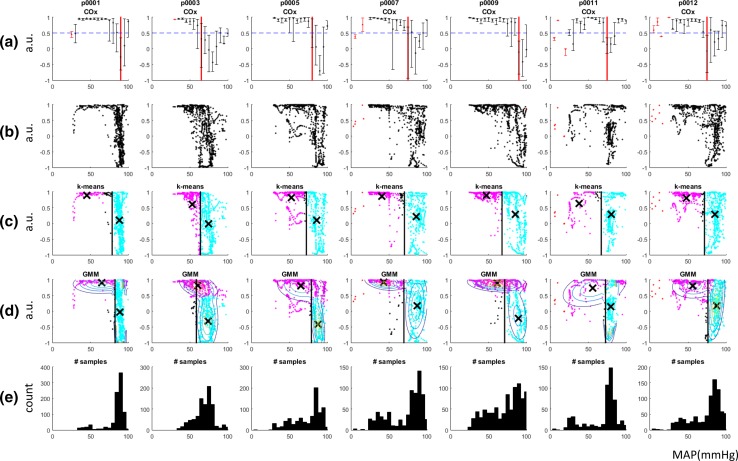
COx results. **a** binned COx plots with the LLA and threshold marked by the *red vertical line* and the *blue horizontal line* respectively. **b** raw COx values. **c** k-means clustering results with the clusters *coloured magenta* and *cyan* (*black* points have been marked as *outliers*), the centroids are marked with a *black* ‘x’ and the LLA with the *black vertical line*. **d** GMM clustering results. **e** Histogram of the number of samples collected at each MAP

Figure 5a contains the traditional COx plots for the seven animals in the study. The calculated LLAs are shown on the plots as red vertical lines. These were determined using the binned data algorithm described above. Bins with too few samples (<5) were excluded from the analysis. These are denoted by red error bars in the plots. The LLAs determined for this binned COx method are given in Table 1. Figure 5b contains the raw, unbinned data corresponding to the plots of Fig. 5a. From visual inspection of these plots, it can be seen that the data all exhibit a tendency towards a COx of unity at low blood pressure indicating impaired autoregulation and a wider distribution across COx values at higher blood pressures corresponding to intact autoregulation.

**Table 1:** LLA’s determined by each method (mmHg)

Data	Algorithm		
	Original binned	k-means	GMM
p0001	90	79	83
p0003	65	64	60
p0005	80	72	79
p0007	75	70	70
p0009	90	68	71
p0011	75	67	73
p0012	75	72	76
Mean	78.6	70.3	73.1
S.D.	8.3	4.4	6.7

Figure 5c shows the k-means clustering of the COx data with the associated LLA’s superimposed (values given in Table 1). The centroids of the k-means clusters are also displayed. The LLAs determined using the algorithm described above are superimposed on the plots as vertical boundaries. Figure 5d shows the Gaussian mixture model clusters of the COx values. The contours of the Gaussian mixtures are superimposed and, again the corresponding LLA’s are overlaid (values also given in Table 1). Figure 5e plots the histograms of the blood pressures visited during each run.

Table 1 contains the LLAs determined by each of the three methods (original binned, k-means, Gaussian mixture model). The mean differences between the original binned data and the k-means and GMM methods are 8.3 and 5.5 mmHg respectively.

Note that a separate data run was performed with the hypo and hyperventilation (which are known to have an effect on autoregulation) portions of the signal removed, however this did not significantly change the COx plots and so all the data was left in the results reported here for completeness.

## Discussion

In practice, the accurate identification of the intact and impaired regions, and hence the LLA point is non-trivial due to the variability of the data both on an inter- and intra-patient basis. Noise may affect the signal in a clinical environment which may be device specific (e.g. amplifier noise, poor sensor positioning, arterial line flushing) or physiological in nature (e.g. caused by the administration of drugs, hypo- or hyper-volemia, hypo- or hyper-ventilation, haemodynamic shifts cause by positional changes, haemorrhage, localised movement). The traditional COx measure using binned data makes the task more difficult due to the associated granularity imposed on the data prior to assessment of the LLA point. It is interesting to note that 14 of the 15 studies referenced in this paper use the ICM+ (Cambridge Enterprise, Cambridge, UK) software [2–9, 11–13, 15–18] which provides the binned autoregulation plots found in much of the scientific literature. The authors, however, advocate the use of new schemes such as the clustering methods described here which may, in fact, provide useful alternatives to autoregulation data analysis and interpretation. The clustering methods employed in the work described here, do not suffer from the imposed granularity of the binned data, and have been shown in this work to identify distinct regions that may be associated with impaired and intact autoregulation zones. The work has obvious wider applicability to other correlation-based metrics for CA, both those derived from NIRS-based technologies (e.g. HVx) and other modalities (e.g. LDx [7], Mx [2], PRx, Fix [18], etc.).

One disadvantage of the study was that the results could not be compared directly to an independent reference signal as historical data was used where no reference signal existed for blood flow or intracranial pressure (as the original study was not set up to investigate autoregulation). However, the cluster-calculated LLA’s could be compared with a standard binned algorithm for determining the LLA from a COx measure. Although this may appear less than ideal, it is worth noting that many of the reference signals that are used in other studies suffer from their own issues. For example TCD, which is required to calculate the Mx measure, requires manual intervention and can be very noisy [6]. In the current study the LLA’s determined through the clustering methods were compared to a standard binned algorithm. Relatively good agreement between the methods was found.

Further tuning is possible for each method, such as changing the COx threshold for the original binned algorithm or modifying the distance metric for the k-means. However, this was not carried out here as it would effectively be an exercise in over-tuning the methods on a small number of subjects. We argued in Sect. 2.2 that a high threshold value should be chosen so that it is distanced from the relatively noisy values around zero corresponding to intact CA and is moved closer to the relatively tightly spread values around unity for impaired CA. For example, increasing the detection threshold to 0.65 results in an even closer relationship between the methods. Specifically, this reduced the LLAs for p0001, p0007 and p0012, resulting in a mean difference between the original binned data and the k-means and GMM methods of 4.0 and 1.5 mmHg respectively (cf. 8.3 and 5.5 mmHg for a threshold of 0.5). However, a full parametric study of the effect of this and other algorithmic modifications would be required in practice to fully hone the technique. This would require a much larger data set for use in a rigorous training and testing framework to prevent over-fitting.

Note that we have focused on the determination of the LLA in this work as the blood pressures associated with the study did not transit the ULAs in any of the animal data. However, in practice in order to find both the LLA and ULA it is trivial to modify this approach to fit three clusters. Additionally, silhouettes [19] could be used as a quality metric—the silhouette measure quantifies how well the data fits the number of clusters—this could be used to provide an indication of confidence in the LLA found or, could be used to switch the algorithm between 2 clusters and 3 clusters in order to automatically switch from LLA only detection to LLA and ULA detection.

The inspection of the cluster plots of Fig. 5 has led the authors to believe that this kind of display may prove advantageous in a clinical setting as it is more intuitive as the standard binned data COx format. In addition, the centroids of the intact regions (also plotted in Fig. 5 as ‘X’s) for all the animals appears to be within a reasonable range for organ autoregulation and could perhaps be used for blood pressure management (e.g. as a target blood pressure). It should also be noted that this clustering technique is not specific to the COx measure, but could be used for other standard correlation measures, e.g. Mx, PRx, HVx, etc.

## Conclusions

We have proposed data clustering methods as a novel strategy for determining and delineating the regions of interest in CA data. Both k-means and Gaussian mixture models were employed to successfully partition COx data into the lower impaired region and intact region, with the boundary at the LLA. These new methods may be considered an alternative possible path towards a NIRS-based continuous autoregulation monitoring algorithm for use in noisy environments such as those encountered in clinical practice. Future work will focus on improving the methods by examining larger data sets where the COx results can be compared to reference Mx data obtained from cerebral blood flow measurements.
